# Abnormalities in electroencephalographic microstates among violent patients with schizophrenia

**DOI:** 10.3389/fpsyt.2023.1082481

**Published:** 2023-02-10

**Authors:** Ruoheng Lin, Qiguang Li, Ziwei Liu, Shaoling Zhong, Qiaoling Sun, Huijuan Guo, Hui Cao, Xiangbin Zhang, Yuhang Hu, Jiansong Zhou, Xiaoping Wang

**Affiliations:** ^1^National Center for Mental and Psychological Diseases Clinical Medical Research Center, The Second Xiangya Hospital of Central South University, Changsha, China; ^2^Department of Psychiatry, The Second Xiangya Hospital of Central South University, Changsha, China; ^3^Xi’an Mental Health Center, Xi’an, China; ^4^School of Medicine, Hunan Normal University, Changsha, China; ^5^Department of Community Mental Health, The Affiliated Brain Hospital of Guangzhou Medical University, Guangzhou, China; ^6^Department of Psychiatry, The Second People’s Hospital of Hunan, Changsha, China; ^7^Department of Neurology, Xiangya Hospital, Central South University, Changsha, China; ^8^Medicine School, Changsha Social Work College, Changsha, China

**Keywords:** resting-state, EEG, microstate, schizophrenia, violence

## Abstract

Schizophrenia is often associated with a remarkably increased risk of violence, which has become a public health concern and brought a great economic burden. Recent studies have reported changes in the electroencephalograms (EEG) of patients with schizophrenia. The evidence for an association between EEG and violence in patients with schizophrenia is not conclusive. This study aimed to investigate EEG microstates in violent patients with schizophrenia. Forty-three violent patients with schizophrenia (the VS group) and 51 non-violent patients with schizophrenia (the NVS group) were included, and their EEG microstates were recorded using 21-Channel EEG recordings. The two groups were compared for differences of four microstate classes (A–D) with regards to three microstate parameters (duration, occurrence, and coverage). Compared with the NVS group, the VS group exhibited increased duration, occurrence, and coverage of microstate class A and decreased occurrence of microstate class B. The VS group also had lower probabilities of transitions from “B to C” and from “C to B”, as compared with the NVS group. In addition, the MOAS score was positively correlated with the duration, occurrence, and coverage of microstate A. The present study found an abnormal pattern of EEG microstates in violent patients with schizophrenia, which might help clinicians identify patients with schizophrenia who might engaged in violence as well as develop intervention strategies at an early stage.

## Introduction

Schizophrenia is associated with an increased risk of violent behavior (1), which has made it a serious public health concern. This mental disorder can also result in a great socio-economic burden (2). Senior et al. estimated that 53% of violent crimes in England and Wales in 2015–2016 were committed by people with serious mental illnesses, which resulted in an annual cost of £2.5 billion (3). Despite the great harm caused by schizophrenia, to date, there is still a lack of knowledge about the neurobiological foundation of violent crime among individuals with schizophrenia.

The identification of potential biomarkers of violent behaviors in schizophrenia is important for the prevention and identification of violence. A large number of functional magnetic resonance imaging (fMRI) studies have reported changes in the structure and function of the brain in violent patients with schizophrenia. Studies have showed that patients with schizophrenia who had a history of violence have reduced volumes in their hippocampus and frontal lobes (especially the orbitofrontal and anterior cingulate cortex) (4) and lower functional connectivity between the amygdala and ventral prefrontal cortex regions (5). Although many fMRI studies have explored the changes in patients with schizophrenia who had engaged in violence, these studies were unable to assess changes in the brain dynamically. This gap can be bridged by electroencephalography (EEG), as EEG has excellent temporal resolution (approximately 1 ms) (6). By recording the electrical potential in the brain using electrodes on the scalp, EEG is able to assess the neural activities and functions of large-scale cortical networks, as well as to capture dynamic and rapid changes (7). Previous studies have found that violent patients with schizophrenia showed a significant increase in slow wave power in the temporal region of the left hemisphere (8), an overall decrease in the alpha power in the occipital and temporal regions, an increase in delta and theta power in bilateral occipital regions, and an increase in delta power in the left temporal region (9). All the above findings have suggested that EEG might be able to differentiate violent and non-violent individuals.

As a quantitative method, EEG microstate analysis can characterize the temporal dynamics of large-scale cortical activities (10). Hence, it can partially compensate for the drawback of EEG in identifying spatial resolution and analyzing large-scale brain network abnormalities. The EEG microstates represent the coordinated activity between cerebral cortices, producing a global pattern of consistent signals across the scalp electrodes; this electric field maintains a steady state at the millisecond level, which is a general representation of the transient electric field of the whole brain (11). EEG microstates have a similar underlying neurophysiological mechanism as fMRI resting state networks (RSNs) (12). However, compared with the spontaneous fluctuation period of slow RSNs of about 10 s in the fMRI, the EEG microstates can help obtain more information (6), as each class of EEG microstate lasts for 80–120 ms and then rapidly switches to another class (13, 14). The time series may provide information on the neural activity of the brain at rest (15–17). Microstate class A reflects the auditory network and is closely associated with changes in blood oxygen level-dependent (BOLD) activation in bilateral temporoparietal and parietal cortices. Microstate class B reflects the visual system and is associated with negative BOLD activation in bilateral occipital cortices. Microstate class C reflects the default network and is associated with positive BOLD activation in dorsal anterior cingulate cortex, bilateral inferior frontal cortex, and right insula regions. Microstate class D reflects the attentional network and is associated with changes in BOLD activation in the right dorsal and ventral regions of frontal and parietal cortices (12, 16). Some researchers believe that the microstates represent emanating mental activities, and collectively produce conscious mental activities and represent the basic building blocks of consciousness. Therefore, microstates were regarded as the smallest unit of cognition and therefore named as “the atom of thought” (6).

A number of studies have found differences in resting-state EEG microstates between patients with schizophrenia and healthy individuals. Cruz et al. found that patients with schizophrenia and their siblings showed an increased presence of microstate class C and a decreased presence of microstate class D (18). One of our previous studies found that patients with schizophrenia exhibited an increased duration and coverage of microstate class C and decreased coverage and incidence of microstate class B, as compared with healthy controls (19). There is a growing body of evidence supporting that schizophrenia with violence might be a biologically based subtype of schizophrenia with neurobiological (20, 21) and neuropsychological deficits (22), and a study has classified it into a different phenotype (23). However, to our knowledge, there has been few studies exploring EEG microstates for violence among patients with schizophrenia. Therefore, the present study looked to investigate the EEG microstate pattern specific to violent patients with schizophrenia, with the hypothesis that the EEG microstate pattern might be different between violent and non-violent patients with schizophrenia.

## Materials and methods

### Participants

Patients with schizophrenia who were undergoing forensic psychiatric evaluation at the Department of Forensic Psychiatry of the Second Xiangya Hospital from January 2013 to December 2016 were recruited. All the enrolled patients met the following inclusion criteria: (1) aged 18–50; (2) with an IQ above 70; and (3) diagnosed with schizophrenia based on the semi-structured interview according to the International Classification of Diseases, 10th edition (ICD-10). Each participant was evaluated by at least two forensic experts. Patients who met the following criteria were excluded: (1) with severe medical or surgical conditions, history of neurological disorders or unstable psychiatric illnesses; (2) with substance abuse or intellectual disability; (3) having used antipsychotics or antidepressants within the last 4 weeks, or any medication within 6 h prior to the examination; and (4) having received electroconvulsive shock or repeated transcranial magnetic stimulation. Based on the presence of history of violent crimes (i.e., commission of intentional homicide, attempted intentional homicide, robbery, arson, explosion, etc.), 43 patients were divided into the group of violent patients with schizophrenia (the VS group), and 51 patients with schizophrenia who were not involved in obvious violent criminal cases (i.e., committing theft, fraud, embezzlement, etc.) were divided into the group of non-violent patients with schizophrenia (the NVS group). All the participants provided written informed consent. The study was approved by the Ethics Committee of the Second Xiangya Hospital.

### Socio-demographic and clinical assessment

Socio-demographic and clinical information was collected using self-designed standardized forms, including gender, age, duration of illness, employment status, level of education, residential status, marital status, psychiatric symptoms, and incidents of aggression.

Overall psychiatric symptoms were assessed using the Brief Psychiatric Rating Scale (BPRS), including anxiety-depression, withdrawal, thought disorder, activation, and hostility-suspiciousness, with a total score of >35 indicating an acute episode of schizophrenia and higher scores indicating higher severity of illness. Current aggression was assessed using the Modified Overt Aggression Scale (MOAS) (24). Items in MOAS are rated on a 5-point Likert scale and covers four behavioral dimensions, including verbal violence, physical violence against objects, violence against self, and violence against others. The total score of MOAS ranges from 0 to 40, with higher scores indicating higher levels of violence.

### EEG recording

A 21-conductor digital paperless EEG device (HSYS-REC-LT2) manufactured by Stellate System Incorporation of Canada was used for the EEG recording in this study. An international 10–20 EEG system was used with electrode impedance kept below 10 KΩ. During the process of EEG recording, the participants sat in a comfortable position with eyes closed, stayed as calm and relaxed as possible in a relatively sound- and light-insulated room, and were asked to open their eyes briefly for 6 s every 3 min to avoid drowsiness. The rate of EEG data recording was 200 Hz, with a filtered bandpass of 0.05–100 Hz, and resting-state EEG was recorded for 5–6 min with participant’s eyes closed.

### Data preprocessing

The raw EEG data were analyzed offline using Matlab (version 2015b) and EEGLAB (version 14.1.2). The EEG data were filtered using a bandpass of 0.05–100 Hz and a notch (49–51 Hz), and were then divided into 2s segments, with eye and muscle movement artifacts removed using the Automatic Artifact Removal toolbox (AAR) in EEGLAB. The data were re-referenced to the common average reference and bandpass filtered (2–20 Hz).

### Microstate analysis

Microstate analysis of the preprocessed EEG data was performed using EEGLAB version 14.1.2 and the Microstates toolkit.^1^ for details The microstate map of each participant was generated using four pre-defined clusters. The number of repetitions was set at 50. The k-means clustering algorithm was used for the microstate analysis using full individual data, with the extraction of the topographies at the global field power (GFP) peaks and removal of polarity. The microstate classes were identified for the VS and NVS groups, respectively. Individual- and group-level maps were fitted using the average microstate classes of all participants as a template, and the following parameters were extracted for the four microstate classes based on the subjects’ average microstate maps: global explained variance, duration (average duration of microstates in milliseconds), coverage (proportion of time spent in each microstate), and occurrence (total number of microstates per second).

### Statistical analysis

Statistical data were analyzed using SPSS version 23.0. The VS and NVS groups were compared separately with regard to basic demographic and clinical characteristics using independent samples *t*-test or chi-square test, as appropriate. Repeated measures analysis of variance (rm-ANOVA) was used for inter-group comparison of microstate parameters between the VS and NVS groups, where microstate parameters (coverage, occurrence, and duration) and microstate classes (classes A, B, C, and D) were used as within-subject factors and group (VS and NVS) was used as a between-subjects factor. Multivariate test with Billet Tracks was applied for multiple comparisons. If significant main effects or interactions were found in the rm-ANOVA, univariate ANOVA would be performed to investigate the simple effects. The transition probabilities were analyzed using one-way ANOVA with Bonferroni correction. Spearman rank correlation analysis was used to analyze the relationship between the MOAS score and the microstate parameters. All analyses were two-sided with *P* < 0.05 regarded as statistically significant.

## Results

### Subject characteristics

With regard to demographic data, no inter-group differences were found in gender, age, level of education, marital status, and employment status (all *P* > 0.05). However, the violent group was more likely to live in rural areas than the non-violent group (*P* = 0.036). Regarding clinical variables, no significant inter-group difference was found in the total score of BPRS (*P* > 0.05). However, the VS group had a significantly higher MOAS score, as compared with the NVS group. The MOAS score of the VS group was 17.05 ± 3.75, significantly higher than 2.16 ± 3.55 in the NVS group (*P* < 0.001). Details are presented in Table 1.

**TABLE 1:** Demographic and clinical characteristics of the VS and NVS groups.

	The VS group (*n* = 43)	The NVS group (*n* = 51)	t/χ^2^	*P*
Gender (M/F, *n*)	35/8	40/11	0.127	0.721
Age (years, mean ± SD)	34.70 ± 7.53	34.90 ± 9.04	−0.118	0.907
Duration of disease (years, mean ± SD)	8.40 ± 6.55	8.25 ± 6.60	0.103	0.918
Marital status (unmarried/married, *n*)	36/7	37/14	1.678	0.195
Compulsory education (no/yes)	28/15	26/25	1.907	0.167
Employment status (unemployed/employed, *n*)	38/5	44/7	0.092	0.761
Residential status (rural/urban, *n*)	38/5	36/15	4.405	0.036
BPRS score	49.21 ± 3.65	48.45 ± 5.76	0.774	0.441
MOAS score	17.05 ± 3.75	2.16 ± 3.55	19.744	<0.001
GEV (%)	70.76 ± 8.81	73.15 ± 7.92	1.384	0.170

VS, violent patients with schizophrenia; NVS, non-violent patients with schizophrenia; BPRS, Brief Psychiatric Rating Scale; MOAS, Modified Overt Aggression Scale.

### Comparison of microstate parameters between the VS and NVS groups

**FIGURE 1:**
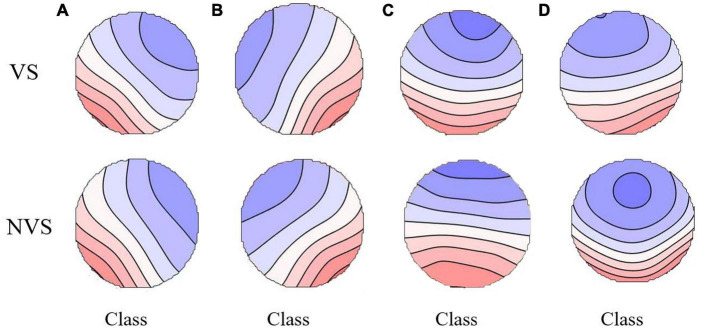
The spatial configuration of the four microstate classes of the two groups. VS, violent patients with schizophrenia; NVS, non-violent patients with schizophrenia.

**FIGURE 2:**
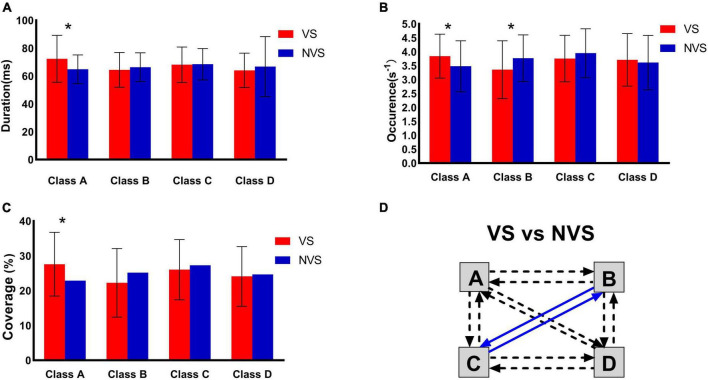
Inter-group comparison of microstate parameters of the VS and NVS groups. **(A)** Duration, **(B)** occurrence, **(C)** coverage, and **(D)** transition probability. VS, violent patients with schizophrenia; NVS, non-violent patients with schizophrenia. *Indicates significant difference (*P* < 0.05). Solid arrows indicate significant correlation. Blue arrows indicate a lower probability in violent patients with schizophrenia.

The maps for the VS and NVS groups are presented in Figure 1. The average global explained variance (GEV) was 70.76 ± 8.81% and 73.15 ± 7.92% for the VS group and the NVS group, respectively, with no significant difference between the two groups (*P* = 0.170). Rm-ANOVA revealed an interaction of microstate parameters × microstate classes × group (F _(6, 87)_ = 3.868, *P* = 0.002). Post hoc analysis showed that the interaction was related to the difference between microstate classes A and B. Compared with the NVS group, patients in the VS group showed an increase in the duration (*P* = 0.009), occurrence (*P* = 0.044), and coverage (*P* = 0.011) of microstate class A, as well as a decrease in the occurrence of microstate B (*P* = 0.037). No statistically significant inter-group differences were found in microstates classes C and D. The VS group had significantly lower probability of transitions from “C to B” (*P* < 0.001), and “B to C” (*P* < 0.001), as compared with the NVS group (Figure 2). The means and standard deviations of all concerned parameters and microstate classes are represented in Supplementary Table 1, detailed results of the rm-ANOVA are presented in Supplementary Table 2, post hoc results are presented in Supplementary Table 3, and transition probabilities in the VS and NVS groups are presented in Supplementary Table 4.

### Relationship between microstate parameters and violence in schizophrenia

The result showed that the MOAS score was positively correlated with the duration (*r* = 0.290, *P* = 0.005), occurrence (*r* = 0.230, *P* = 0.025), and coverage (*r* = 0.295, *P* = 0.004) of microstate A. Details are presented in Table 2.

**TABLE 2:** Correlation for microstate parameters and MOAS score.

	*r*	*P*
Duration A	0.290	0.005
Occurrence A	0.230	0.025
Coverage A	0.295	0.004

## Discussion

To our knowledge, this is the first study to explore EEG microstates in violent patients with schizophrenia, which might provide insight into changes in brain activities in this patient population. Consistent with our hypothesis, the pattern of EEG microstates in violent patients with schizophrenia was significantly different from that in non-violent patients. Although this is only a preliminary study, it has demonstrated that resting-state EEG microstates can be an indicator of violence in patients with schizophrenia.

The present study found that violent patients with schizophrenia had an increased duration, coverage and incidence of microstate class A, and the MOAS score was positively correlated with the duration, occurrence, and coverage of microstate A, indicating that the microstate class A might be associated with an increased risk of violence in schizophrenia. This finding is consistent with previous study, which suggested that violent offenders exhibited a higher level of activation in the network involving the prefrontal and temporoparietal regions (25). Britz et al. found that class A microstates were localized in the bilateral superior temporal gyrus and middle temporal gyrus and were associated with the activation of speech processing in the auditory network (12). Schizophrenia has been known as a highly disabling mental illness with great impact on patients’ family and the society and often manifests as a variety of disorders in perception, thoughts, emotion and behavior. Studies have found that 60–80% of people with schizophrenia may experience hallucinations (26–28). Functional imaging studies have found hyperactivity in the auditory cortex (29, 30) and disruption of activity in the auditory-speech network (31) in patients with command auditory verbal hallucinations. Abnormal connection was also found in language-related areas of the brain in patients with auditory verbal hallucinations (32). Under the control of auditory verbal hallucination, patients may become aggressive in an abrupt manner, leading to self-injurious or violent acts (33, 34). In the present study, the violent patients with schizophrenia showed a significant increase of microstate class A, indicating that this patient population might have more serious abnormal activation of the auditory network.

The present study also found that violent patients with schizophrenia had a reduced occurrence of microstate class B. It has been found that microstate class B is localized in the bilateral visual cortex and occipital cortex, and is extensively connected to the visual network (35, 36), resulting in altered visualization (37). Our results were also in line with findings from prior works. An et al. found that the violence in patients with schizophrenia might be associated with changes in their spatial vision (38). Storvestre et al. found folding and cortical thinning of the visual cortex in violent patients with schizophrenia, indicating impaired visual processing in such patients (39). Moreover, a growing number of studies have found impaired facial emotion recognition in violent patients with schizophrenia (40, 41), which might also be related to altered visual processing (42). Thus, we speculated that the reduction in microstate class B might be associated with impaired visual processing in the visual cortex of violent patients with schizophrenia.

In the present study, no significant differences were found in microstate class C or D. However, some previous studies have found abnormalities in the default and executive networks in violent patients with schizophrenia. Microstate class C has been found to be associated with the default network, which involves medial prefrontal, parietal, and temporal cortices (43). This network is activated during passive resting states or internally oriented mental processes, and is involved in autobiographical memory, theory of mind, and self-referential processing (44). Microstate class D is associated with signals from the right dorsal and ventral regions of the frontal and parietal cortices, which roughly correspond to central executive networks (12). Thijssen et al. reported abnormalities in the default network structure in violent children, specifically, thinner cortices of the default network in violent boys and thicker cortices of the default network in violent girls (45). One of our previous studies showed that male adolescents who had engaged in violent offenses were associated with abnormal functional connectivity in the default mode network (46). Several studies suggested that impaired executive network connectivity was associated with violence and aggression. Karlsgodt et al. found that lower executive function was associated with aggression (47). The disparities might be attributed to different sampling and grouping methods across studies.

The present study also suggested that the probability of transition between microstate classes was different between the VS and NVS groups. Violent patients with schizophrenia showed significantly lower transition probability from “C to B” and “B to C”, as compared with those without a history of violence. As microstate classes are associated with their corresponding functional networks, the probability of transition between microstate classes may indicate the switching between different functional networks (12, 17). Changes in transition probabilities between microstate classes might reflect alterations of network connection. The findings of the present study suggest an alteration in the connectivity between the visual network and the default network in violent patients with schizophrenia.

### Limitation

Some limitations need to be mentioned in this study. Firstly, the relatively small sample size might have led to possible biases. Secondly, although the participants in the present study had received treatments, they had not used medication within 4 weeks prior to the start of the study, thus, the impact of medication was little. Thirdly, due to the limitation of research conditions, healthy non-violent and healthy but violent individuals were not included, which might have impacted our findings to some extent. Recruitment of such individuals is ongoing to expand the dataset for further analysis.

## Conclusion

Violent patients with schizophrenia were found to have specific changes in EEG microstates. This finding may have significant implications for the assessment and management of violence in this patient population. However, due to the nature of this pilot study, the findings need to be interpreted with caution. Future studies with larger sample sizes are needed to further investigate microstates in violent patients with schizophrenia.

## Data availability statement

The original contributions presented in this study are included in the article/Supplementary material, further inquiries can be directed to the corresponding author.

## Ethics statement

Studies involving human subjects are reviewed and approved by the Second Xiangya Hospital of Central South University. The participant’s legal guardian/next of kin signed the written informed consent for this study.

## Author contributions

XW and JZ conceived and designed the research and revised the manuscript. RL contributed to the data collection and analysis and wrote the first draft of the manuscript. QL, ZL, SZ, QS, HG, HC, XZ, and YH contributed to the data collection. All authors have approved the final manuscript.

## Footnotes

1
http://www.thomaskoenig.ch/index.php/software/microstates-in-eeglab
