# Supplementary material for: Abnormalities in electroencephalographic microstates among violent patients with schizophrenia
Source: Front Psychiatry. 2023 Feb 10;14:1082481. doi: 10.3389/fpsyt.2023.1082481 (PMC9950110; doi:10.3389/fpsyt.2023.1082481)
Supplement: Supplementary file 1 [file Data_Sheet_1.docx]

**Supplementary Tables**

**Supplementary Table 1.** Means and standard deviations of all considered parameters and microstate classes.

|  | | Group | |
| --- | --- | --- | --- |
|  |  | VS | NVS |
| Duration (ms) | A | 72.38±16.81 | 64.83±10.32 |
|  | B | 64.46±12.47 | 66.39±10.33 |
|  | C | 68.12±12.73 | 68.49±11.26 |
|  | D | 64.07±12.38 | 66.79±21.54 |
| Occurrence (s^-1^) | A | 3.84±0.79 | 3.48±0.91 |
|  | B | 3.36±1.04 | 3.77±0.84 |
|  | C | 3.75±0.84 | 3.95±0.88 |
|  | D | 3.71±0.94 | 3.61±0.98 |
| Coverage (%) | A | 27.61±9.15 | 22.91±8.37 |
|  | B | 22.25±9.84 | 25.14±7.67 |
|  | C | 26.03±8.64 | 27.3±8.68 |
|  | D | 24.11±8.58 | 24.65±10.97 |

VS, violent patients with schizophrenia; NVS, non-violent patients with schizophrenia.

**Supplementary Table 2**. Results of rm-ANOVA on microstate temporal parameters.

| rm-ANOVA | F | *P* |
| --- | --- | --- |
| MS | 167111.709 | < 0.001 |
| MS × Group | 0.115 | 0.892 |
| PAR | 1.367 | 0.258 |
| PAR × Group | 5.594 | 0.001 |
| PAR × MS | 0.941 | 0.470 |
| PAR × MS × Group | 3.868 | 0.002 |

MS, microstate class; PAR, microstate parameter; Billet Tracks was applied for multiple comparisons.

**Supplementary Table 3.** Post-hoc inter-group comparisons of all microstate parameters (duration, occurrence, and coverage), with Bonferroni correction for all microstate classes (A, B, C, and D).

| Microstate parameters | Microstate class | VS vs. NVS | | |
| --- | --- | --- | --- | --- |
|  |  | *P* | d | 95%CI |
| Duration (ms) | A | 0.009 | 7.552 | 1.9316 - 1.317 |
|  | B | 0.412 | -1.936 | -6.606 - 2.733 |
|  | **C** | 0.883 | -0.366 | -5.282 - 0.455 |
|  | D | 0.466 | -2.718 | -10.097 - 4.661 |
| Occurrence (s^-1^) | A | 0.044 | 0.364 | 0.010 - 0.717 |
|  | B | 0.037 | -0.409 | -0.793 - -0.025 |
|  | C | 0.276 | -0.195 | -0.548 - 0.158 |
|  | D | 0.615 | 0.100 | -0.295 - 0.496 |
| Coverage (%) | A | 0.011 | 4.701 | 1.108 - 8.294 |
|  | B | 0.113 | -2.891 | -6.480 - 0.698 |
|  | C | 0.481 | -1.269 | -4.830 - 2.293 |
|  | D | 0.793 | -0.542 | -4.634 - 3.551 |

VS, violent patients with schizophrenia; NVS, non-violent patients with schizophrenia.

**Supplementary Table 4. Transition probabilities in the VS and NVS groups.**

|  | VS (43) | | NVS (51) | |  |  |  |
| --- | --- | --- | --- | --- | --- | --- | --- |
|  | Mean | SD | Mean | SD | t | *P* | Comparison |
| A-B | 0.0819 | 0.0399 | 0.0779 | 0.0334 | 0.529 | 0.598 | VS>NVS |
| A-C | 0.0961 | 0.0509 | 0.0788 | 0.0332 | 1.982 | 0.050 | VS>NVS |
| A-D | 0.0735 | 0.0178 | 0.0672 | 0.0355 | 1.060 | 0.292 | VS>NVS |
| B-A | 0.0819 | 0.0411 | 0.0791 | 0.0315 | 0.381 | 0.704 | VS>NVS |
| B-C | 0.0592 | 0.0165 | 0.0849 | 0.0284 | -5.476 | < 0.001 | VS＜NVS |
| B-D | 0.0752 | 0.0362 | 0.0782 | 0.0380 | -0.388 | 0.699 | VS＜NVS |
| C-A | 0.0947 | 0.0509 | 0.0791 | 0.0332 | 1.786 | 0.077 | VS>NVS |
| C-B | 0.0598 | 0.0176 | 0.0860 | 0.0285 | -5.443 | < 0.001 | VS＜NVS |
| C-D | 0.0931 | 0.0468 | 0.0907 | 0.0428 | 0.260 | 0.796 | VS>NVS |
| D-A | 0.0743 | 0.0181 | 0.0654 | 0.0361 | 1.469 | 0.145 | VS>NVS |
| D-B | 0.0750 | 0.0361 | 0.0786 | 0.0377 | -0.479 | 0.633 | VS＜NVS |
| D-C | 0.0924 | 0.0469 | 0.0917 | 0.0421 | 0.073 | 0.942 | VS>NVS |

VS, violent patients with schizophrenia; NVS, non-violent patients with schizophrenia.
